# Supplementary material for: Phylogenetics and Population Genetics of the Petrolisthes lamarckii–P. haswelli Complex in China: Old Lineage and New Species
Source: Int J Mol Sci. 2023 Oct 31;24(21):15843. doi: 10.3390/ijms242115843 (PMC10648172; doi:10.3390/ijms242115843)
Supplement: Supplementary file 1 [file ijms-24-15843-s001.zip › ijms-2668169-supplementary.new.pdf]

Table S1. Genbank numbers for 5-gene.

Samples	COI	16S	Cytb	18S	H3
XG_HS1	OR460807	OR466985	OR476711	OR467004	OR476741
XG_HS2	OR460807	OR466985	OR476711	OR467004	OR476741
XG_HS3	OR460807		OR476711		OR476741
XG_HS4	OR460807	OR466985	OR476711		OR476741
XG_HS5	OR460807	OR466985	OR476711		OR476741
XG_HS6	OR460807		OR476711		OR476741
XG_HS7	OR460807		OR476711		OR476741
XG_HS8	OR460807		OR476711		
XG_HS9	OR460807		OR476711		OR476741
XG_HS10	OR460807		OR476711		OR476741
XG_HS11	OR460807	OR466985	OR476711		OR476741
XG_HS12	OR460807	OR466985	OR476711		OR476741
XG_HS13	OR460807		OR476711		
XG_HS14	OR460807	OR466985	OR476711		
XG_HS15	OR460807	OR466985	OR476711		OR476741
XG_HS16	OR460807		OR476711		
XG_HS17			OR476711		OR476741
XG_HS18	OR460807	OR466985	OR476711		OR476741
XG_HS19			OR476711		OR476741
XG_HS20	OR460807	OR466985	OR476711		OR476741
XG_HS21	OR460807	OR466985	OR476711		OR476741
XG_HS22	OR4608010	OR466985	OR476711		
XG_HS23	OR460807	OR466985	OR476711		OR476741
XG_HS24	OR460807	OR466985	OR476711		
XG_HS26	OR460807	OR466985	OR476711		
XG_HS27	OR460807		OR476711		
XG_HS28	OR460807	OR466985	OR476711		OR476741
XG_HS29		OR466985	OR476711		OR476741
XG_HS30	OR460807	OR466985	OR476711		
XG_HS31	OR460807	OR466985	OR476711		
XG_HS32	OR460807	OR466985	OR476711		
XG_HS33	OR460807	OR466985	OR476711		
XG_HS34	OR460807	OR466985	OR476711		OR476741
DS_HS1	OR460807	OR466985	OR476711	OR467004	OR476741
DS_HS2	OR460807	OR466985	OR476711	OR467004	OR476741
DS_HS3	OR460807	OR466985	OR476711	OR467004	OR476741
DS_HS4	OR460807	OR466985	OR476711	OR467004	OR476741

DS_HS5	OR460808	OR466986	OR476711	OR467004	OR476741
DS_HS6	OR460807	OR466985	OR476711	OR467004	OR476741
DS_HS7	OR460807	OR466985	OR476711	OR467004	OR476741
DS_HS8	OR460807	OR466985	OR476711		OR476741
HZ_HS1	OR460807	OR466985			OR476741
HZ_HS2	OR460807	OR466985			
HZ_HS3	OR460807	OR466985			OR476741
HZ_HS4	OR460807	OR466985	OR476711		
HZ_HS5	OR460807	OR466985	OR476711		
HZ_HS6	OR460807	OR466985	OR476711		
HZ_HS7	OR460807	OR466985	OR476711		
HZ_HS8	OR460807	OR466985	OR476711		OR476741
HZ_HS9	OR460807	OR466985	OR476714		
HZ_HS11	OR460807	OR466985	OR476711	OR467004	OR476741
HZ_HS12	OR460807	OR466985	OR476711	OR467004	OR476741
MW_HS1	OR460807	OR466985	OR476711	OR467004	
MW_HS2	OR460807	OR466985	OR476711		
MW_HS3	OR460807	OR466985	OR476711	OR467004	OR476741
MW_HS4	OR460807	OR466985	OR476711		
MW_HS5	OR460807	OR466985	OR476711		
MW_HS6	OR460807	OR466985	OR476711		
FCG_HS1	OR460807	OR466985	OR476711	OR467004	
FCG_HS2	OR460807	OR466985	OR476712	OR467004	OR476741
FCG_HS4	OR460807	OR466985			OR476741
FCG_HS5	OR460807	OR466985			OR476741
WZD_HS1	OR460807	OR466985			OR476741
WZD_HS2	OR460807				
WZD_HS3	OR460807				
WZD_HS5	OR460807	OR466985	OR476711	OR467004	OR476741
WZD_HS6	OR460807	OR466985	OR476711	OR467004	OR476741
WZD_HS7	OR460807				
WZD_HS8	OR460807				
WZD_HS9	OR460807				
WZD_HS10	OR460807				
WZD_HS11	OR460807				
WZD_HS12	OR460807			OR467004	
WZD_HS13	OR460807			OR467004	
XW_HS1	OR460807	OR466985	OR476711	OR467004	OR476741
XW_HS2	OR460807	OR466985	OR476711		
XW_HS3	OR460807				OR476741
XW_HS4	OR460807				

XW_HS5	OR460807				
XW_HS6	OR460807				
XW_HS7	OR460807				
DZ_HS1	OR460807	OR466985	OR476711	OR467004	OR476741
DZ_HS2	OR460807	OR466985	OR476711	OR467004	OR476741
DZ_HS3	OR460807	OR466985			
DZ_HS4	OR460807	OR466985			OR476741
DZ_HS5	OR4608020	OR466994	OR476719	OR467006	OR476743
DZ_HS6	OR4608021	OR466993	OR476720	OR467006	OR476743
DZ_HS7	OR460807	OR466985			OR476741
DZ_HS8	OR460807	OR466985			OR476741
DZ_HS9	OR460807				
DZ_HS10	OR460807				
DZ_HS11	OR460807				
DZ_HS12	OR460807				
DZ_HS13	OR4608018	OR466993	OR476721	OR467006	OR476743
DZ_HS14	OR4608019	OR466993	OR476722	OR467006	OR476743
JJD_HS1	OR4608011	OR466988		OR467005	OR476742
JJD_HS2	OR4608012	OR466989	OR476715	OR467005	OR476742
JJD_HS5	OR4608013	OR466990	OR476716	OR467005	OR476742
JJD_HS6	OR460807				
JJD_HS7	OR4608014	OR466991	OR476717	OR467005	OR476742
JJD_HS8	OR4608015	OR466992	OR476715	OR467005	OR476742
SYHS_HS1	OR4608033	OR466995	OR476737	OR467007	OR476744
SYHS_HS2	OR4608024	OR466995	OR476738	OR467007	OR476744
SYHS_HS3	OR4608034	OR466995	OR476739	OR467007	OR476744
LHT_HS1	OR4608022	OR466995	OR476726	OR467007	OR476744
LHT_HS2	OR4608018	OR466993	OR476723	OR467006	OR476743
LHT_HS3	OR4608030	OR466996	OR476728	OR467007	OR476744
LHT_HS4	OR4608024	OR466995	OR476727	OR467007	OR476744
LHT_HS5	OR4608024	OR466995	OR476726		OR476744
LHT_HS6	OR4608024	OR466997			OR476744
LHT_HS7	OR460809	OR466987	OR476713	OR467004	OR476741
LHT_HS8	OR4608032	OR466995	OR476729		OR476744
LHT_HS9	OR4608024	OR466998		OR467007	OR476744
LHT_HS10	OR4608018	OR466993	OR476720	OR467006	OR476743
LHT_HS11	OR4608023	OR466995	OR476730	OR467007	OR476744
LHT_HS12	OR4608024	OR466995	OR476731	OR467007	OR476744
LHT_HS13	OR4608016	OR466989		OR467005	OR476742
LHT_HS14	OR4608024	OR466999	OR476732		OR476744
LHT_HS15	OR460807	OR466985	OR476711	OR467004	OR476741

LHT_HS16	OR460807	OR466985	OR476711	OR467004	OR476741
LHT_HS17	OR4608024	OR466995		OR467007	OR476744
LHT_HS18	OR4608014	OR466989	OR476715	OR467005	OR476742
LHT_HS19	OR4608017	OR466989	OR476718	OR467005	OR476742
LHT_HS20	OR4608024	OR467000		OR467007	OR476744
LHT_HS21	OR460808	OR466986	OR476711	OR467004	OR476741
LHT_HS22	OR4608025	OR467001	OR476733	OR467007	OR476744
LHT_HS23	OR4608026	OR466995	OR476734		OR476744
LHT_HS24	OR4608027	OR466995			OR476744
LHT_HS25	OR4608028	OR466999	OR476726	OR467007	OR476744
LHT_HS26	OR4608029	OR467002	OR476735	OR467007	OR476744
LHT_HS27	OR4608018	OR466993	OR476725	OR467006	OR476743
LHT_HS28	OR460807	OR466985	OR476711		OR476741
LHT_HS29	OR4608024	OR466995			OR476744
LHT_HS30	OR4608031	OR466995	OR476736	OR467007	OR476744
LHT_HS31	OR4608018	OR466993	OR476724	OR467006	OR476743
Australia_1	NC025572	NC025572	NC025572		
Pisidia serratifrons	OR460835	OR467003	OR476740	OR467008	OR476745
Pisidia magdalenensis		MN715756	MN712001	MN715573	MN712187
P_AH135	KY857297	KY857020	MN711994	MN715566	MN712180
P_AHG16A	KY857428	KY857151			
P_AHU19A	KY857525	KY857248	MN711996	MN715568	MN712182
P_AHO11A	KY857520	KY857243	MN711997	MN715569	MN712183

Table S2. The number of all dataset and mito dataset obtained through Phylosuit.

Location	Group	Taxon	Mito dataset	All dataset
Xiaguan	XG	*P. haswelli*	10	1
Dongshan	DS	*P. haswelli*	8	2
Huizhou	EC	*P. haswelli*	8	2
Miaowan		*P. haswelli*	6	1
Xuwen	BH	*P. haswelli*	2	1
Fangchenggang		*P. haswelli*	2	1
Weizhoudao		*P. haswelli*	2	2
Danzhou		*P. haswelli*	2	2
		P. polychaetus	4	4
Jiajingdao	HN	*P. haswelli*	1	0
		P. lamarckii	4	4
Sanyahouhai		*P. shanyingi* sp. nov.	3	3
Luhuitou		*P. haswelli*	5	4
		P. polychaetus	4	4
		P. lamarckii	2	2
		P. shanyingi sp. nov.	13	9

Table S3. Best-fit model according to BIC of ML and Bayesian tree.

Data	ML Tree	Bayesian Tree
COI	TIM2+F+G4	
16S	K3Pu+F+G4	
Cytb	TPM3+F+I	
18S	K2P	
H3	HKY+F+I	
Mito dataset	TPM2u+F+G4: CO1,<br>K3Pu+F+G4: 16S,<br>TPM3+F+G4: CYTB.	
All dataset	TPM2u+F+G4: CO1,<br>K3Pu+F+I: 16S,<br>K2P+I: 18S+H3,<br>TPM3u+F+I+G4: CYTB.	GTR+F+G4: CO1,<br>HKY+F+I: 16S,<br>K2P+I: 18S+H3,<br>HKY+F+G4: CYTB.

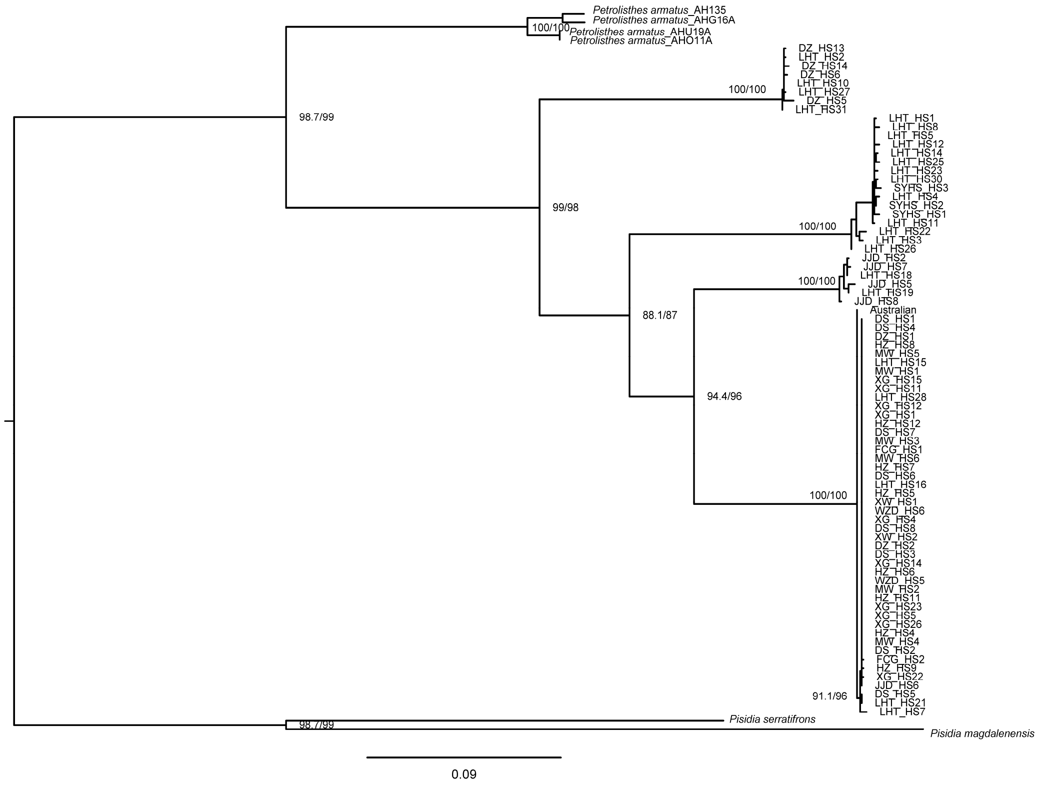

Figure S1. Maximum likelihood phylogeny of *Petrolisthes* derived from a concatenated mitochondrial (16S, COI and Cytb) dataset using IQ-Tree.

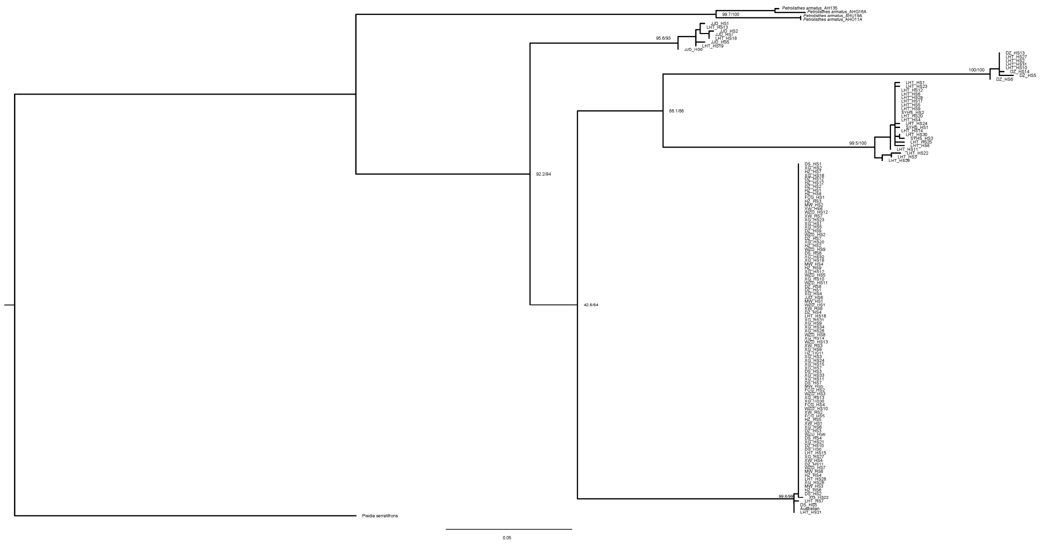

Figure S2. Maximum likelihood phylogeny of *Petrolisthes* derived from COI dataset using IQ-Tree.

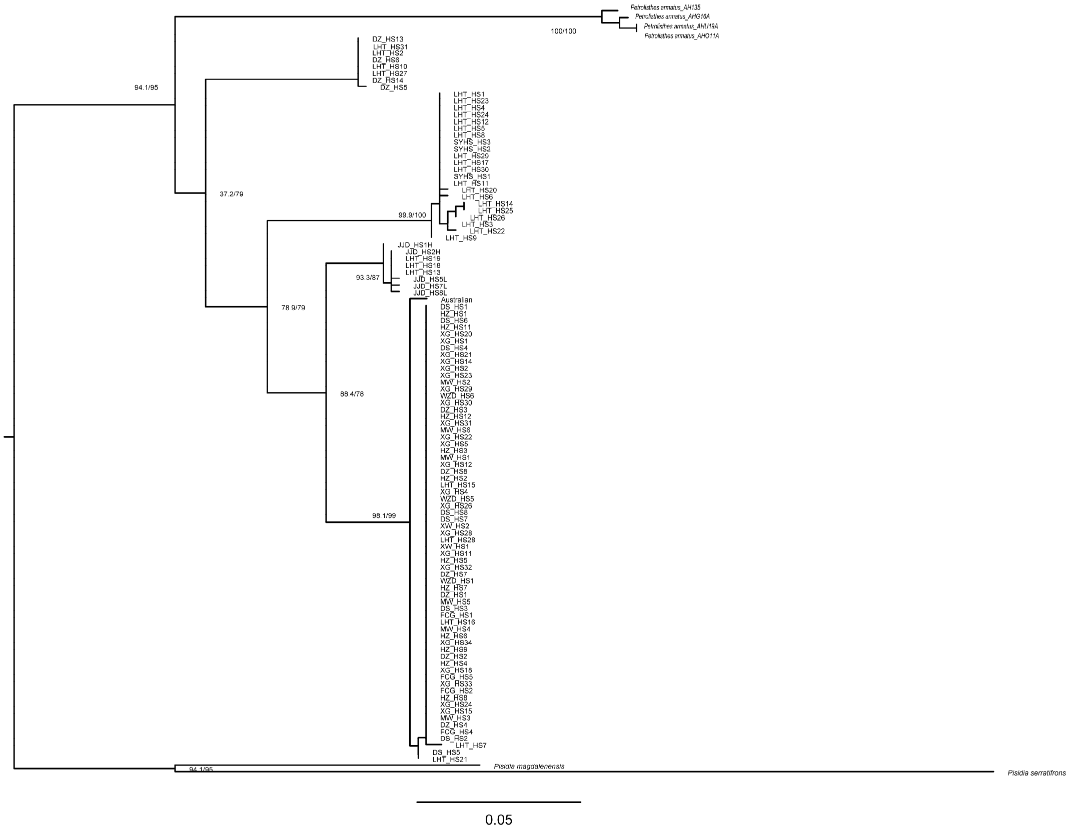

Figure S3. Maximum likelihood phylogeny of *Petrolisthes* derived from 16S dataset using IQ-Tree.

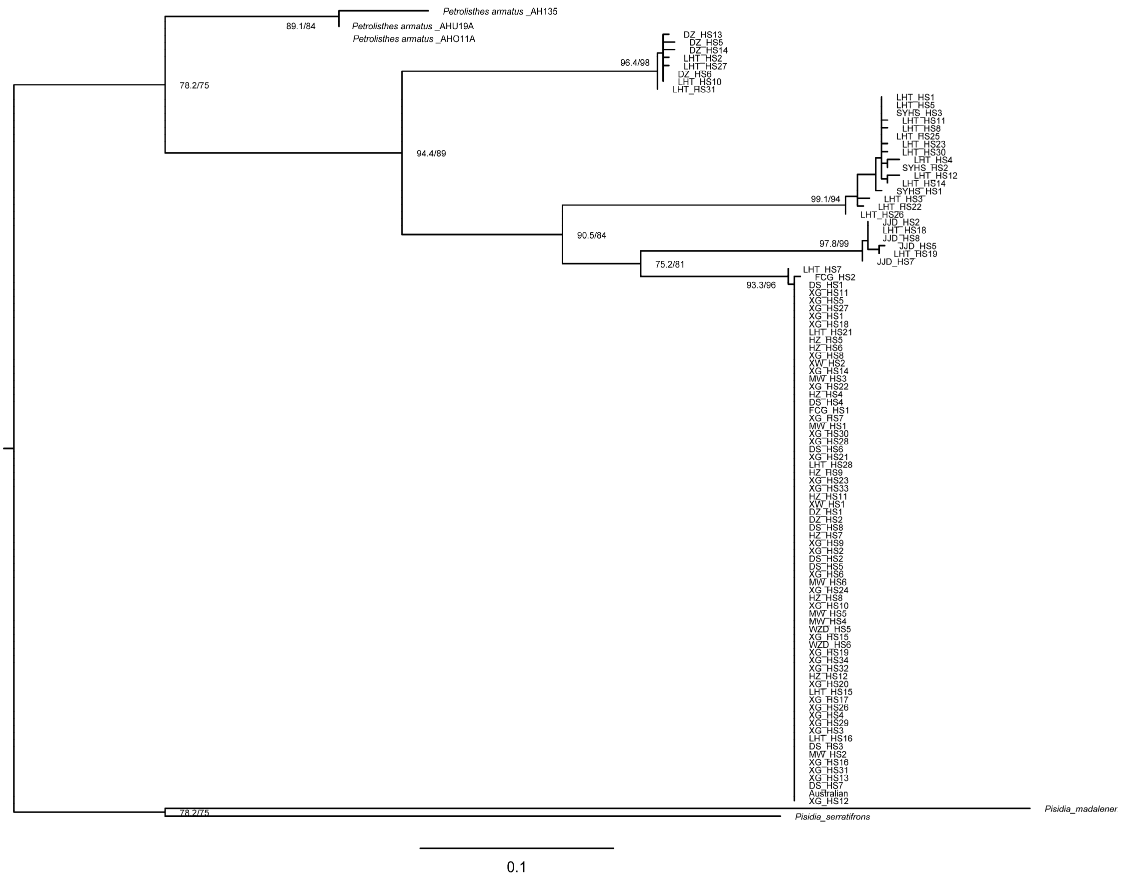

Figure S4. Maximum likelihood phylogeny of *Petrolisthes* derived from Cytb dataset using IQ-Tree.

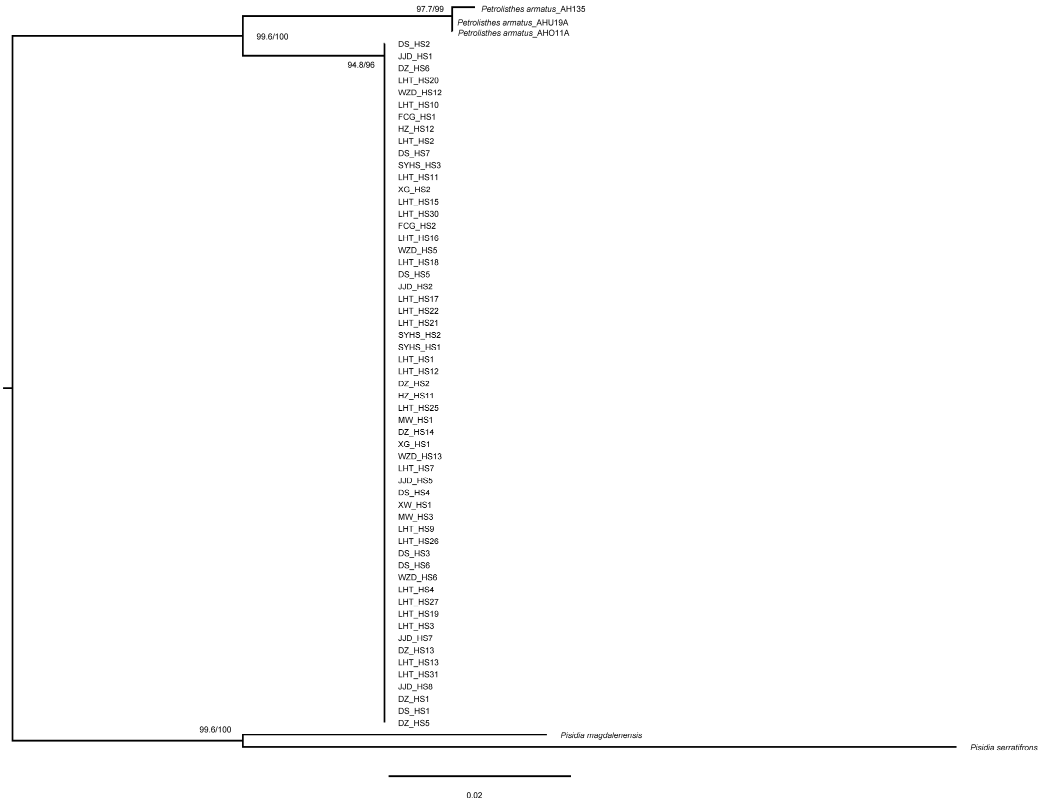

Figure S5. Maximum likelihood phylogeny of *Petrolisthes* derived from 18S dataset using IQ-Tree.

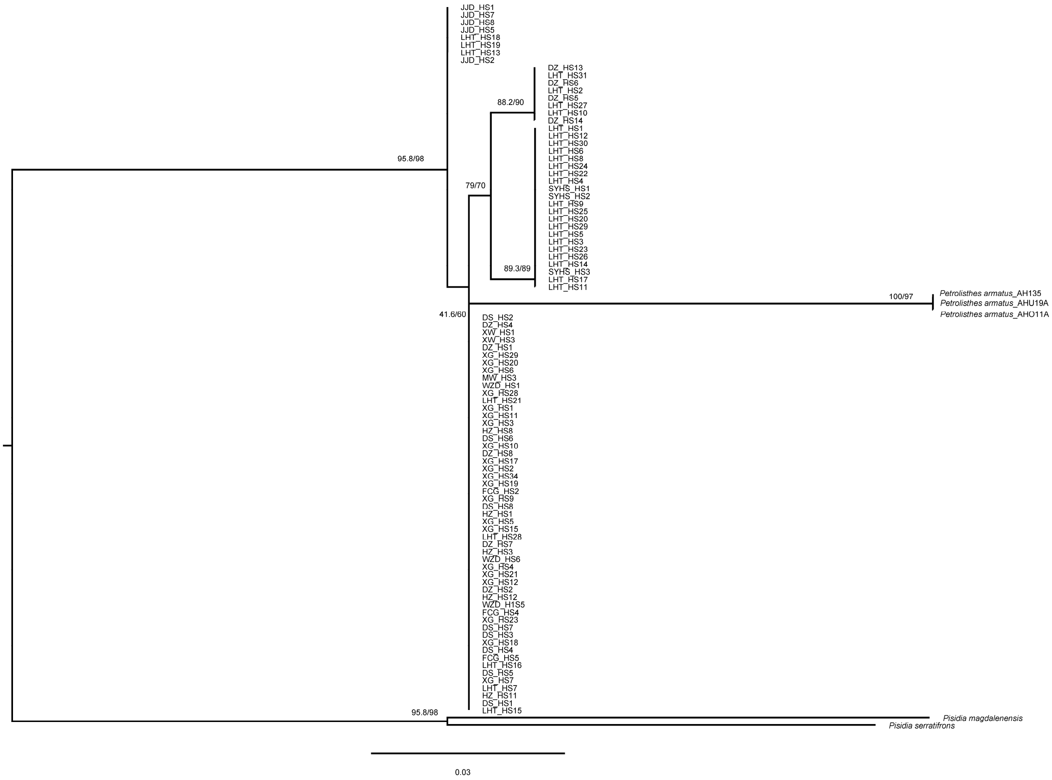

Figure S6. Maximum likelihood phylogeny of *Petrolisthes* derived from H3 dataset using IQ-Tree.

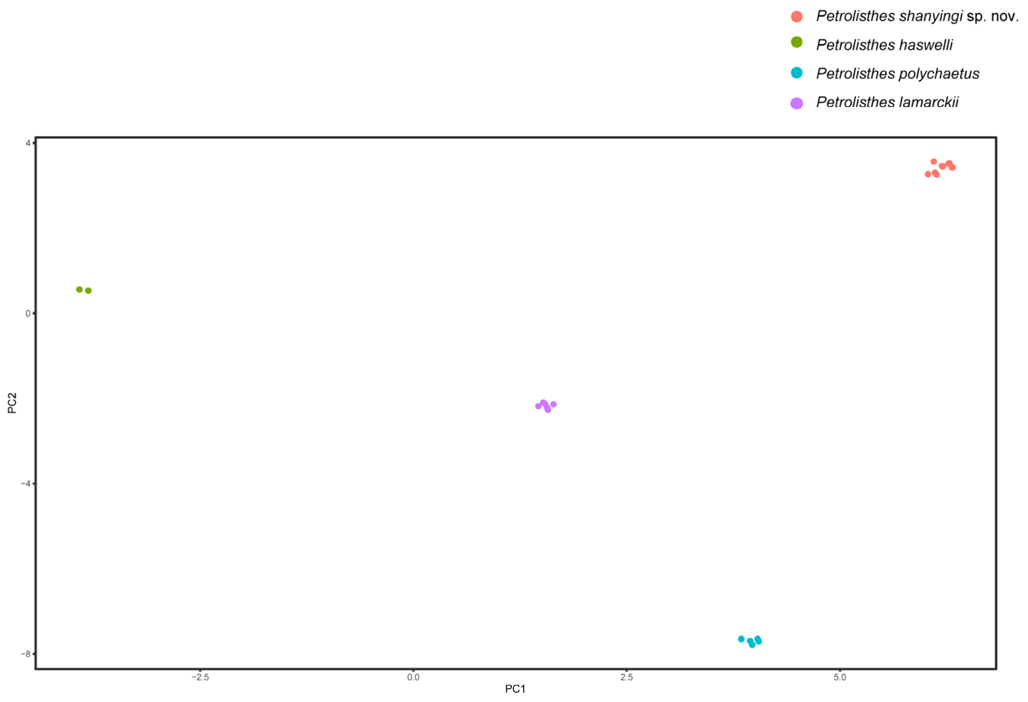

Figure S7. Scatterplot generated by analysis of principal components of *Petrolisthes* based on mito dataset among lineages.
